# Uncovering the pharmacological mechanisms of Zizhu ointment against diabetic ulcer by integrating network analysis and experimental evaluation *in vivo* and *in vitro*

**DOI:** 10.3389/fphar.2022.1027677

**Published:** 2022-12-13

**Authors:** Jie Wang, Yu Wang, Renyan Huang, Wenhui Li, Weijing Fan, Xiaoming Hu, Xiao Yang, Qiang Han, Hongfei Wang, Guobin Liu

**Affiliations:** ^1^ Department of Peripheral Vascular Surgery, Shuguang Hospital Affiliated to Shanghai University of Traditional Chinese Medicine, Shanghai, China; ^2^ Academy of Integrative Medicine, Shanghai University of Traditional Chinese Medicine, Shanghai, China; ^3^ Collaborative Innovation Center, Shanghai University of Medicine and Health Sciences, Shanghai, China; ^4^ Beicai Community Health Service Center, Shanghai, China

**Keywords:** Zizhu ointment, diabetic ulcer, PI3K-AKT signaling pathway, macrophage, network analysis, UPLC-HRMS

## Abstract

Diabetic ulcer (DU) has been recognized as one of the most prevalent and serious complications of diabetes. However, the clinical efficacy of standard treatments for DU remains poor. Traditional Chinese medicine (TCM) shows a positive therapeutic effect on DU. Specifically, Zizhu ointment (ZZO) has been widely used to treat DU in long-term clinical practice, but the exact mechanism by which it promotes DU wound healing remains unknown. In this study, network analysis and high-performance liquid chromatography–high resolution mass spectrometry (UPLC-HRMS) were conducted to identify the active compounds of ZZO. We detected isovalerylshikonin (ISO), mandenol, daidzein, kaempferol, and formononetin in both network analysis and UPLC-HRMS. Moreover, ZZO could ameliorate DU by regulating the phosphatidylinositol-3-kinase (PI3K)/protein kinase B (AKT) and inflammation signaling pathways, according to the results of KEGG analysis. We established a DU mouse model with a high-fat diet and streptozotocin injection *in vivo* to evaluate the network analysis result. The experimental results showed that ZZO could inhibit inflammation, remodel fibrous tissue, and promote angiogenesis in the DU area, facilitating wound healing in DU mice. Moreover, the PI3K/AKT signaling pathway was indeed activated by ZZO treatment, promoting macrophage M2 polarization. In addition, we used molecular docking technology to evaluate the binding sites between ZZO and the PI3K/AKT pathway. The results showed that ISO has a good binding interaction with AKT. Moreover, ISO promoted M2 polarization in macrophages in a dose-dependent manner *in vitro.* Our study found that ZZO could promote DU wound healing by inhibiting inflammation, which was achieved by macrophage M2 polarization through activating the PI3K/AKT pathway. Further studies have demonstrated that ISO plays major role in the above process. These findings provide a theoretical basis for further preclinical evaluation and lay a foundation for nano-gel compound treatment with ZZO.

## Introduction

Diabetic ulcer (DU) is regarded as one of the most prevalent complications of diabetes, with a global prevalence of 6.3% (Gupta et al., 2017), leading to high mortality and disability in diabetes patients (Zhao et al., 2020). About 19–34% of diabetes patients may develop a DU during their lifetime (Armstrong et al., 2017). Moreover, the recurrence rates of DU remain high, at approximately 40% within one year and 65% within five years (Armstrong et al., 2017). Clinically, the current treatment of DU includes debridement, antibiotics, and revascularization, but its efficacy is poor (da Silva et al., 2010; Shang et al., 2019). Thus, it is urgent to find an effective additional therapy to promote the wound healing of DU when combined with standard treatment.

Traditional Chinese medicine (TCM) has become popular in the treatment of DU at multiple levels and in multiple pathways (Zhou et al., 2022). In a systematic review of 49 randomized controlled trials of TCM anti-DU, Wang et al. found that healing rates with TCM therapy were 42–60.4%, or twice times as high as healing rates with standard therapy (Wang et al., 2019). Our research group has been engaged in clinical studies of TCM in anti-DU for a long time. We have found that Zizhu ointment (ZZO)—which consists of cinnabaris (Zhusha, ZS), *Astragalus mongholicus* Bunge (Fabaceae; Huangqi, HQ), *Arnebia guttata* Bunge (Boraginaceae; Zicao, ZC), *Asini corii colla* (Ejiao, EJ), Borneolum (Bingpian, BP), and *Calamus draco* Willd. (Arecaceae; Xuejie, XJ)—is helpful in clinical practice (Huang et al., 2022). We recruited 76 DU patients to compare wound healing rates with ZZO treatment and basic fibroblast growth factor (bFGF) spay treatment. The results showed that ZZO is more effective in DU recovery, with a response rate of up to 50% compared with 28% in the control group (Han and Liu, 2021). Recently, the preparation process, parameters, and clinical efficacy of ZZO have been awarded a national invention patent (*ZL201010186284.X*).

**FIGURE 1:**
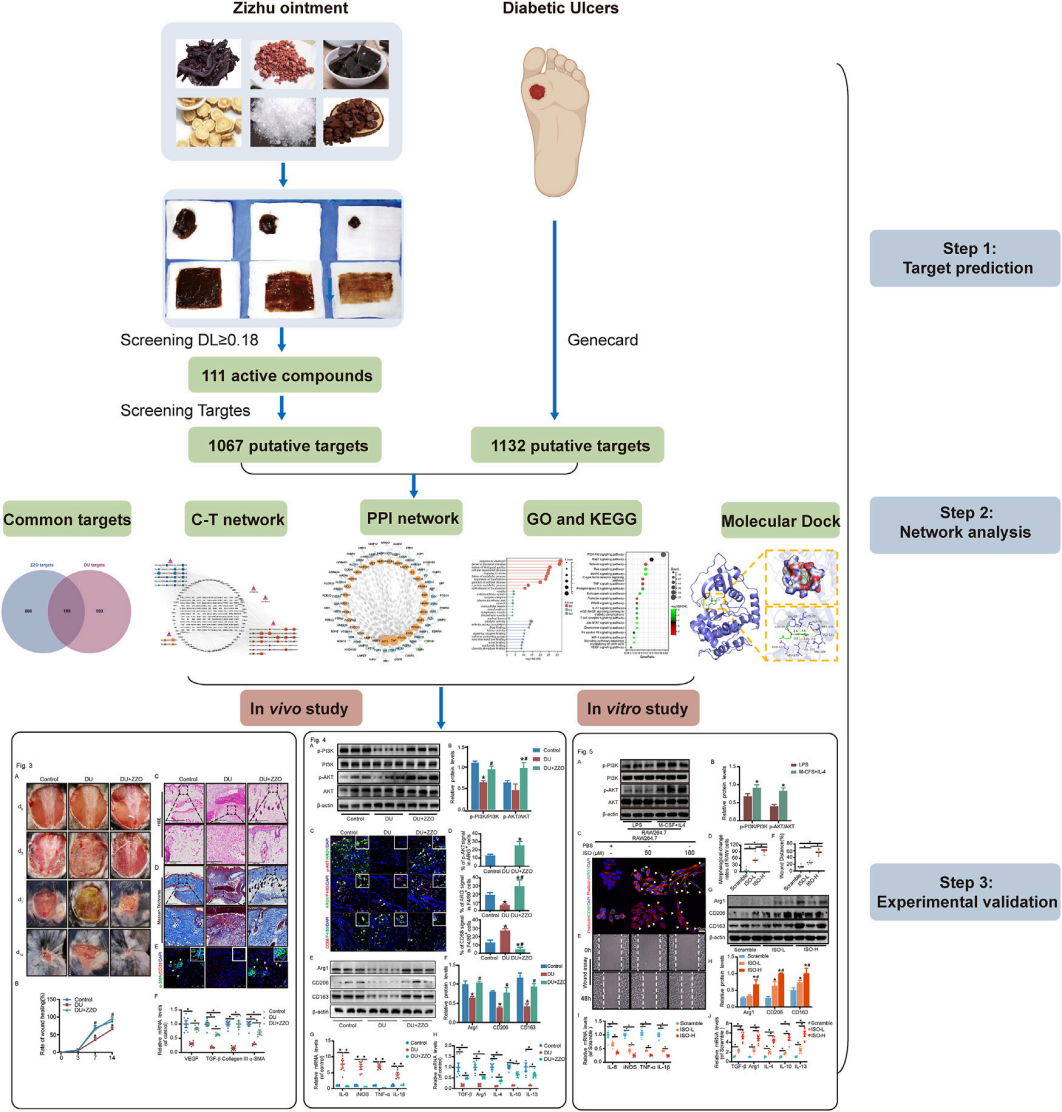
Graphical summary of this study.

Based on the accepted anti-DU clinical efficacy of ZZO, we aimed to further investigate its mechanisms and active compounds. Our previous study found that ZZO accelerates the wound healing process *via* inhibiting Notch4 signaling and promoting the M2 polarization of macrophages (Huang et al., 2022). Nevertheless, the anti-DU active ingredients of ZZO have not been clearly elucidated and warrant further exploration. In this study, we adopted a network analysis approach, employing the framework of “one drug, one target, one disease” (Gocho et al., 2021) to understand the mechanisms underlying ZZO against DU. We also used high-performance liquid chromatography–high resolution mass spectrometry (UPLC-HRMS) to compare the compounds screened from the network analysis. Furthermore, the mechanisms of ZZO predicted by network analysis were evaluated *in vivo* and *in vitro*. An overview flowchart of the study design is shown in Figure 1.

## Materials and methods

### Composition and preparation of ZZO and chemical component profiling

The crude ingredients of ZZO were purchased from Shanghai Kangqiao Chinese Medicine Tablet Co., Ltd. and processed by the Wuhan Ma Yinglong workshop, with all ingredients corresponding to quality control standards. The ratios of the main medicines (by weight) of ZS, ZC, XJ, HQ, EJ, and BP were 7: 3: 3: 6: 5: 1, and the ratio of the components of ZZO to the base materials was 1: 8 (Huang et al., 2022). The ZZO preparation was also composed of excipients including poloxamer, propylene, water, glycerol, polyethylene glycol (PEG), PEG 1500, PEG 4000, and ethyl p-hydroxybenzoate, for which the ratios were 34: 24: 70: 2.6: 9:9: 3:0.06.

### UPLC-MS analysis

UPLC-MS analysis was accomplished by Shanghai Applied Protein Technology. Purified ZZO samples of 0.02 g were weighed and placed in 1.5 ml centrifuge tubes. The samples were dissolved in 1 ml of 70% methanol, then vortexed for 30 min and centrifuged (16,000 g/15 min, 4°C, Eppendorf Centrifuge 5430 R). The supernatant was collected and vacuum freeze-dried. The residue after lyophilization was again dissolved in 2 ml of 40% methanol. The samples were vortex mixed, rotated, and centrifuged (16,000 g/15 min, 4°C), and the supernatant was collected. The ZZO extracts were analyzed using a UPLC-HRMS system (UPLC, ACQUITY Waters UPLC HSST3; MS, Q Exactive, Thermo Scientific). UPLC separation was conducted on an ACQUITY UPLC HSS T3 column (2.1 × 100 mm, 1.8μm, Thermo Scientific) at a flow rate of 0.3 ml/min. HPLC-grade solvents and additives were from ThermoFisher Scientific (United States). The gradient program using 0.1% formic acid in water (phase A) and 0.1% formic acid acetonitrile (phase B) was adopted as follows: 95% A at 0 min to 2% A at 17 min, 95% A at 17.2 min, lasting for 2.8 min, then back to original condition. The column temperature was 35°C and the injection volume of ZZO solution was 2 μL. MS analysis was conducted using the positive ion mode. The instrument was calibrated using external standards before analysis to ensure a mass accuracy of better than 3 ppm throughout the experiment. The source parameters were as follows: spray voltage of 3.8 kV, capillary temperature of 320°C, sheath gas flow rate of 45 arb. units, Aux gas flow rate of 20 arb. units, spare gas flow rate of 0 arb. units, and probe heater temperature of 370°C. A full MS scan (m/z 90–1300) with a resolution of dd-MS2 was used.

## Analysis of ZZO by network analysis

### Prediction of the active compounds in ZZO

The candidate compounds of ZZO were collected from the Traditional Chinese Medicine Systems Pharmacology Database and Analysis Platform (TCMSP) (http://tcmsp-e.com.tcmsp.php), the largest database to include data for TCM pharmacology (Ru et al., 2014), and the Herbal Ingredients’ Targets Database (HIT) database (http://lifecenter.biosino.org/hit/; Xiao et al., 2019). As ZZO is not absorbed orally, drug-like quality (DL), an established concept used in drug design to evaluate the solubility and chemical stability of a potential compound, was adopted as a parameter for the initial screening of the active compounds (Jia et al., 2020). Herein, we adopted a DL value ≥0.18 as the criterion for identifying prospective active compounds (Li et al., 2020).

### Potential common targets of ZZO against DU

Multiple ingredients of ZZO exerted interactive synergistic therapeutic effects with multiple targets. We used the Swiss Target Prediction (http://www.swisstargetprediction.ch; Gfeller et al., 2014) and PharmMapper databases (http://lilab-ecust.cn/pharmmapper/; Gu et al., 2020) and the STITCH system (http://stitch.embl.de/cgi/input.pl; Aihaiti et al., 2021) to screen potential targets of these compounds. Meanwhile, known DU-related targets were screened by the DrugBank database (https://www.drugbank.ca/; Wishart et al., 2018), the online Mendelian Inheritance in Man (OMIM) database (https://omim.org/search/advanced/geneMap), and the GeneCards database (https://www.genecards.org/) using the keywords “diabetic foot ulcer” and “diabetic ulcer” (Sayers et al., 2021) in the OMIM and GeneCards databases (choosing the target of relevance score >5). (Tao et al., 2020). All potential targets were converted to official names by importing them into the UniProt knowledge base (https://www. uniprot.org/; UniProt Consortium, 2021). The targets common to ZZO and DU were identified as potential therapeutic targets.

### Constructing the common target predicted protein–protein interaction network

To detect the systemic effect of the common target proteins, these targets were imported into the online STRING database (https://string-db.org/; Szklarczyk et al., 2021), which contains confirmed PPI, and the confidence level score was set to ≥0.4. Moreover, Cytoscape software (version 3.9.1) was used to visualize the PPI network (Kohl et al., 2011) and to evaluate the statistical properties of nodes in the network. We included targets with top-100-degree values for further pathway analyses.

### Gene Ontology (GO) and Kyoto Encyclopedia of Genes and Genomes pathway enrichment analyses

The top-100 degree targets were evaluated by GO enrichment and KEGG pathway analysis using the Sangerbox database (https://www.sangerbox.com/), and only items with *p*-values <0.05 were selected (Shen et al., 2022). Furthermore, human disease pathways were excluded, and the gene ratio>10 pathways in KEGG analysis were included.

### Molecular docking

The structures of the compounds were downloaded from the PubChem database (https://pubchem.ncbi.nlm.nih.gov/; Kim et al., 2016). Next, the protein structures were imported into the Chem3D software (Meng et al., 2004), and the 3D crystal structures of compounds were obtained. Meanwhile, the structures of targets were obtained from the Research Collaboratory for Structural Bioinformatics database (https://www.rcsb.org/structure/2ZUT; Segura et al., 2020), including the removal of ligands and water molecules, addition of polar hydrogen, and combination of non-polar hydrogen. Then, the compounds and targets were imported into Schrödinger Maestro software (Friesner et al., 2004). The compounds were shortlisted based on their docking scores in the Standard Precision method.

## Experimental evaluation

### Establishment of a DU mouse model and treatment

Adult male C57BL/6 mice (21–25 g, 8–10 weeks) were purchased from Shanghai Model Organisms Center, Inc. The mice were housed in the Experimental Animal Center of Shanghai University of TCM in a specific pathogen-free environment under a 12 h light/dark cycle with freely available water and food. Mice were randomly assigned to each group (*n* = 4), including the non-diabetic group (phosphate-buffered saline, PBS) and the DU group without or with ZZO treatment. DU groups were fed a high-fat diet comprised of 60% calories (FB-D12451, Wuxi Fan Bo Biotechnology Co., Ltd.) for three weeks and subjected to STZ (40 mg/kg/day, ip. Cat. No. 2196GR001, BioFRoxx) for one week (Cheng et al., 2021). It was considered type 2 diabetes until the level of fasting blood glucose exceeded 11.1 mmol/L (Gupta et al., 2016). Subsequently, full-thickness skin wounds (1*1 cm, with depth to the fascial layer) were surgically created on the back of mice by lifting the skin with forceps (Qiu et al., 2020). The wound was bandaged with 1 cm^2^ gauze with excipients or ZZO (50 mg/cm^2^). The dressing was changed daily. All animal experiments were approved by the Animal Ethics Committee of Shanghai University of Traditional Chinese Medicine (Approval No. PZSHUTCM220711028) and correspond to the National Institutes of Health guide for the care and use of Laboratory animals (NIH Publications No. 8023, revised 1978).

### Wound closure analysis

To assess the condition of wound healing in mouse backs, the wound was photographed by a digital camera (Nikon, Tyoto) on days 0, 3, 7, and 14 (Huang et al., 2020). The wound closure rate was quantified by ImageJ software (Bethesda, MD) and calculated as follows: t-t_0_/t_0_ × 100% (t: the wound healing was assessed, t_0_: initial wounding).

### Hematoxylin and Eosin and Masson staining

The wound tissues were fixed with 4% paraformaldehyde (PFA, Cat. No. P0099, Beyotime) for 48 h. Following standard paraffin embedding and sectioning, the wound tissues were subjected to hematoxylin and eosin (H&E, Cat. No. C01105M, Beyotime, China) and Masson staining (Cat. No. G1340, Solarbio Life Sciences, China). Stained tissues were analyzed using a digital slide scanning system (Precipoint M8).

### Immunofluorescence staining

To detect the co-localization of macrophages, a double IF was performed. After routine deparaffinization, rehydration, and antigen repair, the sections were incubated with 3% bovine serum albumin (BSA; Cat. No.3610 ES76, Yeasen Biotech Co., Ltd.). Then a rabbit anti-F4/80 antibody (Cat. No. 30325, CST; 1:200) mixed with a mouse anti-arginase 1(ARG1; Cat. No.SC-271430, Santa Cruz; 1:200) or a mouse anti-CD68 (Cat. No. YM3050, Immunoway; 1:200), and a mouse anti-ARG1(Cat. No.SC-271430, Santa Cruz; 1:200) mixed with a rabbit anti-p-AKT (Cat. No.9271S, CST; 1:200) were incubated overnight at 4°C. Tissue sections were washed with PBS three times and cultured in the dark with donkey anti-mouse Alexa Fluor 488 (Cat. No. A21202, Life Technologies; 1:500) or donkey anti-rabbit Alexa Fluor 488 (Cat. No. A21206, Life Technologies; 1:500) and donkey anti-rabbit Alexa Fluor 555 (Cat. No. A31572, Life Technologies; 1:500) or goat anti-mouse Alexa Fluor 555 (Cat. No. A31570, Invitrogen; 1:500) for 2 h at room temperature at the next day and then washed three times with PBS. Nuclei were counterstained with DAPI (Cat. No.C1006, Beyotime) for 10 min and then washed with PBS; antifade mountant (Cat. No. P0126, Beyotime) was added to each coverslip, and the coverslips were placed on the slides. Positive signals were captured using a confocal microscope (Leica SP-8, Leica Corporation, German) and further analyzed with the Adobe Photoshop CS software program. Analysis of fibrosis and angiogenesis was performed by dual IF with mouse anti-α-SMA (Cat. No. MA5-15871, Invitrogen; 1:200) and rabbit anti-CD31 antibodies (Cat. No. PA5-32321, Invitrogen; 1:200). The methods were as described previously.

### RNA extraction and quantitative real-time PCR array

Trizol (Cat. No. R0016, Beyotime, China) was used to extract RNA according to the manufacturer’s instructions. NanoDrop ND-1000 was used to determine the concentration of RNA, and the extracted RNA was stored in a refrigerator at −80°C. Using 500 ng total RNA as a template, cDNA was synthesized with the cDNA Synthesis Kit (Cat. No. R312, Vazyme, China). Samples were stored at −20°C and subjected to qPCR using a StepOnePlus Real-Time PCR System (Applied Biosystems). Each qPCR sample was performed in a 10 μL reaction containing 2xSYBR Green qPCR Master Mix (Cat. No. R711-02, Vazyme, China), 10 nM forward and reverse primers, and 2 μL cDNA in triplicate. The qPCR protocol was executed for 45 cycles, with each cycle consisting of denaturation at 95°C for 15 s, annealing at 60°C for 1 min, and extension at 72°C for 1 min. Using *GAPDH* as an internal control, quantitative PCR analysis was performed to quantify the relative mRNA expression of targeted genes. The result of qPCR from the threshold cycle (Ct) and use the 2^−△△Ct^ method was defined to calculate the relative expression level. The primer pairs specific for various genes used in our experiments are listed in Table1.

**TABLE 1:** Prime sequences.

Primer	Forward (5′–3′)	Reverse (5′–3′)
*mGapdh*	TGG​ATT​TGG​ACG​CAT​TGG​TC	TTT​GCA​CTG​GTA​CGT​GTT​GAT
*mArg1*	TGT​GGG​AAA​AGC​CAA​TGA​AC	GGTGTCAGCGGAGTGTTG
*mIl-4*	CGT​GAT​GTA​CCT​CCG​TGC​TT	GTG​AGT​TCA​GAC​CGC​TGA​CA
*mIl-13*	CCT​GGC​TCT​TGC​TTG​CCT​T	GGT​CTT​GTG​TGA​TGT​TGC​TCA
*mIl-10*	TTG​AAC​CAC​CCG​GCA​TCT​AC	CCA​AGG​AGT​TGC​TCC​CGT​TA
*mIl-6*	GCT​ACA​GCA​CAA​AGC​ACC​TG	GAC​TTC​AGA​TTG​GCG​AGG​AG
*miNos*	GAT​AAA​GGG​ACA​GCG​TCA​GC	CCT​TCG​GGC​CAA​AGA​TCC​TG
*mTnf-α*	TAC​TGA​ACT​TCG​GGG​TGA​TTG​GTC​C	CAG​CCT​TGT​CCC​TTG​AAG​AGA​ACC
*mIl-1β*	ATC​TCG​CAG​CAG​CAC​ATC​AAC	TGT​TCA​TCT​CGG​AGC​CTG​TAG​T
*mα-SMA*	GTC​CCA​GAC​ATC​AGG​GAG​TAA	TCG​GAT​ACT​TCA​GCG​TCA​GGA
*m-CollaIII*	CTG​TAA​CAT​GGA​AAC​TGG​GGA​AA	CCA​TAG​CTG​AAC​TGA​AAA​CCA​CC
*m-Vegf*	GAG​GTC​AAG​GCT​TTT​GAA​GGC	CTG​TCC​TGG​TAT​TGA​GGG​TGG
*m-Tgfβ*	ATG​TCA​CGG​TTA​GGG​GCT​C	GGC​TTG​CAT​ACT​GTG​CTG​TAT​AG

### Western blot analysis

Wound tissues and cells were lysed in a RIPA lysis buffer (Cat. No. P0013C, Beyotime, China) containing proteinase and phosphatase inhibitor cocktails (Cat. No. P1005, Beyotime, China; Cat. No. P1045, Beyotime, China). An equal amount of protein sample (20 μg) from each group was loaded on a 7.5% or 10% SDS-PAGE gel (Cat. No. PG111, PG112, EpiZyme, China) along with standard molecular weight markers (Cat. No.26619, 26,625, Thermo Fisher, United States), followed by transfer onto a polyvinylidene difluoride (PVDF) membrane (Cat. No. IPVH00010, Millipore), which was then blocked with 5% BSA for 2 h. Membranes were incubated overnight at 4°C with a rabbit anti-p-AKT antibody (Cat. No. T40067, Abmart; 1:3000), a rabbit anti-p-PI3K antibody (Cat. No. T40064, Abmart; 1:3000), a mouse anti-PI3K antibody (Cat. No. 13666S, CST; 1:2000), a rabbit anti-AKT antibody (Cat. No. 4691S, CST; 1:1000), a rabbit anti-Arg1 antibody (Cat. No. 93668T, CST; 1:2000), a rabbit anti-CD206 antibody (Cat. No. 24595S, CST; 1:2000), a rabbit anti-CD163 antibody (Cat. No. 93498S, CST; 1:2000), and a rabbit anti-β-actin antibody (Cat. No. AF7018, Affinity; 1:5000) in 5% BSA. After rigorously washing with TBS containing 0.1% Tween-20 (Cat. No. T8220, Solarbio, China), membranes were incubated at room temperature for 1 h with a goat anti-rabbit HRP-conjugated IgG antibody (Cat. No. A0208, Beyotime; 1:10,000) or a goat anti-mouse HRP-conjugated IgG antibody (Cat. No. A0216, Beyotime; 1:10,000). Subsequently, membranes were washed three times with TBST. Target proteins were visualized using a super-sensitive electrochemiluminescence (ECL) reagent (Cat. No. MA0186, Meilunbio, China) with a Molecular Imager ChemiDoc XRS System (Tanon).

### Cell culture

RAW264.7 (mouse macrophage line) cells were purchased from American Type Culture Collection and cultured in Dulbecco’s Modified Eagle Medium (DMEM) supplemented with 10% heat-inactivated fetal bovine serum (FBS), 100 U/ml penicillin, and 100 μg/ml streptomycin under 37°C, 5% CO_2_ conditions. M1 macrophages were induced by lipopolysaccharide (LPS; 100 ng/ml, Cat. No. L2630, Sigma-Aldrich, United States; Lv et al., 2021), whereas M2 macrophages were induced by IL-4 (20 ng/ml, Cat. No. 214-14, Peprotech, United States) and M-CSF (25 ng/ml, Cat. No. 315-02, Peprotech, United States) (Moore et al., 2015).

### Cell morphological assay

RAW264.7 cells treated with ISO-L (50 μM, Cat. No. HY-N3012, MCE) or ISO-H (100 μM) were grown on coverslips in 24-well plates to about 30% confluency. After 24 h of incubation, cells were fixed with 4% PFA for 30 min. After washing with PBS three times, cells were stained with a mouse anti-CD68 antibody (Cat. No. YM3050, Immunoway; 1:200) and a mouse anti-ARG1 antibody (Cat. No.SC-271430, Santa Cruz; 1:200) overnight at 4°C. Next, cells were gently rinsed three times with PBS and incubated in the dark with donkey anti-mouse Alexa Fluor 488 (Cat. No. A21202, Life Technologies; 1:500) for 2 h at room temperature. After rigorous rinsing, cells were stained with Alexa Fluor 555-phalloidin (Cat. No. A34055, Invitrogen; 1:200) for 30 min at 37°C. Subsequently, nuclei were counterstained with 4′,6-diamidino-2-phenylindole (DAPI; Cat. No. C1006, Beyotime, China) and mounted with an antifade mounting medium (Cat. No. P0126, Beyotime, China). Photographs were taken using a confocal microscope (Leica SP-8, Leica Corporation, German).

### Wound healing assay

RAW264.7 cells were seeded into a 6-well plate and then treated with ISO-L or ISO-H. After the cells grew to a 100% confluent layer, 2 ml of serum-free DMEM medium was added. A 10μL pipette tip was used to make a straight scratch on the upper surface. Then, cell debris was removed with PBS, and images were taken at 0 and 48 h using an inverted microscope.

### Statistical analysis

Statistical analyses were conducted using GraphPad Prism (GraphPad, United States). One-way ANOVA and two-way ANOVA were used for comparisons between multiple groups, and pairwise comparisons within groups were analyzed by the Student’s *t*-test. *p*-values <0.05 were considered to indicate statistical significance.

## Results

### Identification of active components of ZZO

**FIGURE 2:**
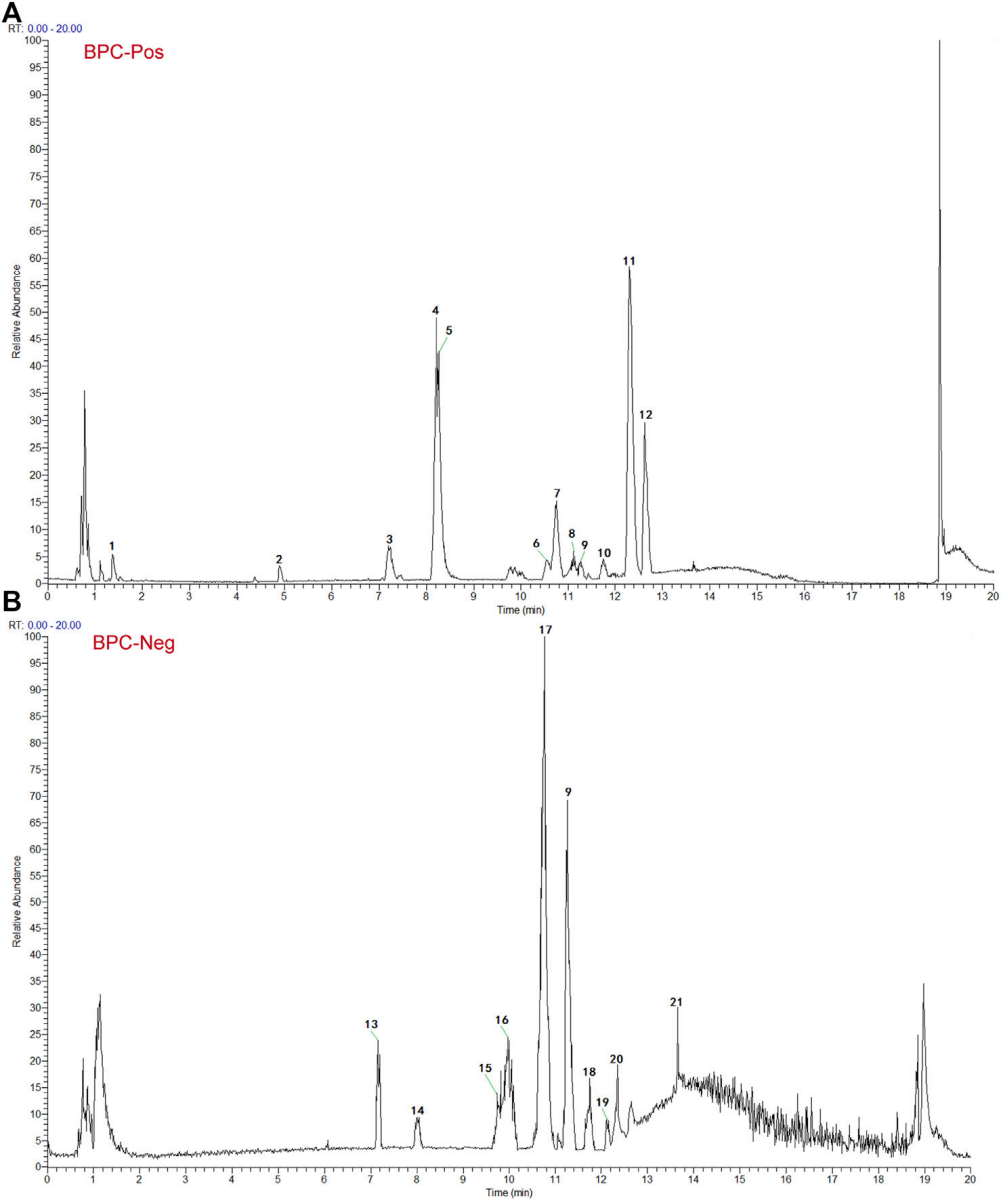
Identification results of the main chemical components of ZZO by UPLC-Q-HRMS. **(A)** Total ion flow diagram of ZZO in negative mode. **(B)** Total ion flow diagram of ZZO in positive ion mode.

Based on the TCMSP and HIT, 111 compounds from ZS, HQ, ZC, EJ, BP, and XJ were obtained. A total of four duplicate compounds were included, including oleanolic acid, rhamnocitrin, daidzein, and formomonetin. Moreover, there were found to be 38 compounds in ZC, 61 compounds in HQ, 5 compounds in BP, 5 compounds in ZS, 4 compounds in EJ, and 2 compounds in XJ. The overall compounds in ZZO are summarized in Supplementary Table S1. Moreover, ZZO was further analyzed by UPLC-HRMS and yielded 392 identified compounds (Supplementary Table S2); the top 21 compounds of ZZO are shown in (Figure 2; Table 2).

**TABLE 2:** The top 21 compounds of ZZO by UPLC-HRMS analysis.

PeakNo	m/z	RT min	ppm	Compound name	Score	PubCHEM	Molecular formula
						CID	
1	155.0818	1.40	0.4	Pyrrolopiperazine-2,5-dione	0.997	Cid_193540	C_7_H_10_N_2_O_2_
2	188.1432	4.92	0.6	N-[1-(2-Phenylethyl)-4-piperidinyl]-2-naphthamide	0.9995	Cid_35296170	C_24_H_26_N_2_O
3	253.0857	7.23	0.2	4′-Methoxyflavone	0.9975	Cid_77793	C_16_H_12_O_3_
4	267.1019	8.22	0.2	3,4′-Dimethoxy-2-hydroxychalcone	0.9938	Cid_5976425	C_17_H_16_O_4_
5	298.2163	8.25	11.4	C11:db-UHQ aka 2-undecenyl-quinoloin-4(1H)-one position of double bond unknown	0.9637	Cid_129846253	C_20_H_27_NO
6	167.0703	10.56	0.9	3,5-Dimethoxycinnamic acid	0.8941	Cid_5324677	C_11_H_12_O_4_
7	285.1122	10.74	0.1	1,2-Propanediol, 1,2-dibenzoate	0.9944	Cid_517637	C_17_H_16_O_4_
8	137.1326	11.13	0.3	Eucalyptol	0.9981	Cid_2758	C_10_H_18_O
9	257.0810	11.28	1.9	Pinocembrin	0.9871	Cid_68071	C_15_H_12_O_4_
10	137.1326	11.77	0.8	Decalin-2-carboxylic acid	0.9977	Cid_656885	C_11_H_18_O_2_
11	372.1167	12.31	17.1	Isovalerylshikonin	0.9895	Cid_479497	C_21_H_24_O_6_
12	271.1329	12.63	21.4	Difenpiramide	0.7295	Cid_100472	C_19_H_16_N_2_O
13	303.2169	7.17	0.3	Aleuritic acid	0.9985	Cid_222178	C_16_H_32_O_5_
14	181.0502	8.02	3.4	Gallacetophenone-4′-methyl ether	0.9889	Cid_12820	C_9_H_10_O_4_
15	269.0814	9.76	0.1	Echinatin	0.915	Cid_6442675	C_16_H_14_O_4_
16	245.3384	9.98	5.3	Mandenol	0.988	Cid_5282184	C_20_H_36_O_2_
17	283.0976	10.74	21.6	Curculigoside	0.8576	Cid_158845	C_22_H_26_O_11_
18	398.3437	11.74	6.81	Daidzein	0.993	Cid_5281708	C_15_H_10_O_4_
19	269.0820	12.13	0.3	Cryptostrobin	0.9949	Cid_6453244	C_16_H_14_O_4_
20	243.1959	12.35	1	14-Hydroxymyristic acid	0.997	Cid_3084276	C_14_H_28_O_3_
21	271.2274	13.68	0.1	3-Hydroxypalmitic acid	0.9972	Cid_301590	C_16_H_32_O_3_

### Compound–target network construction

**FIGURE 3:**
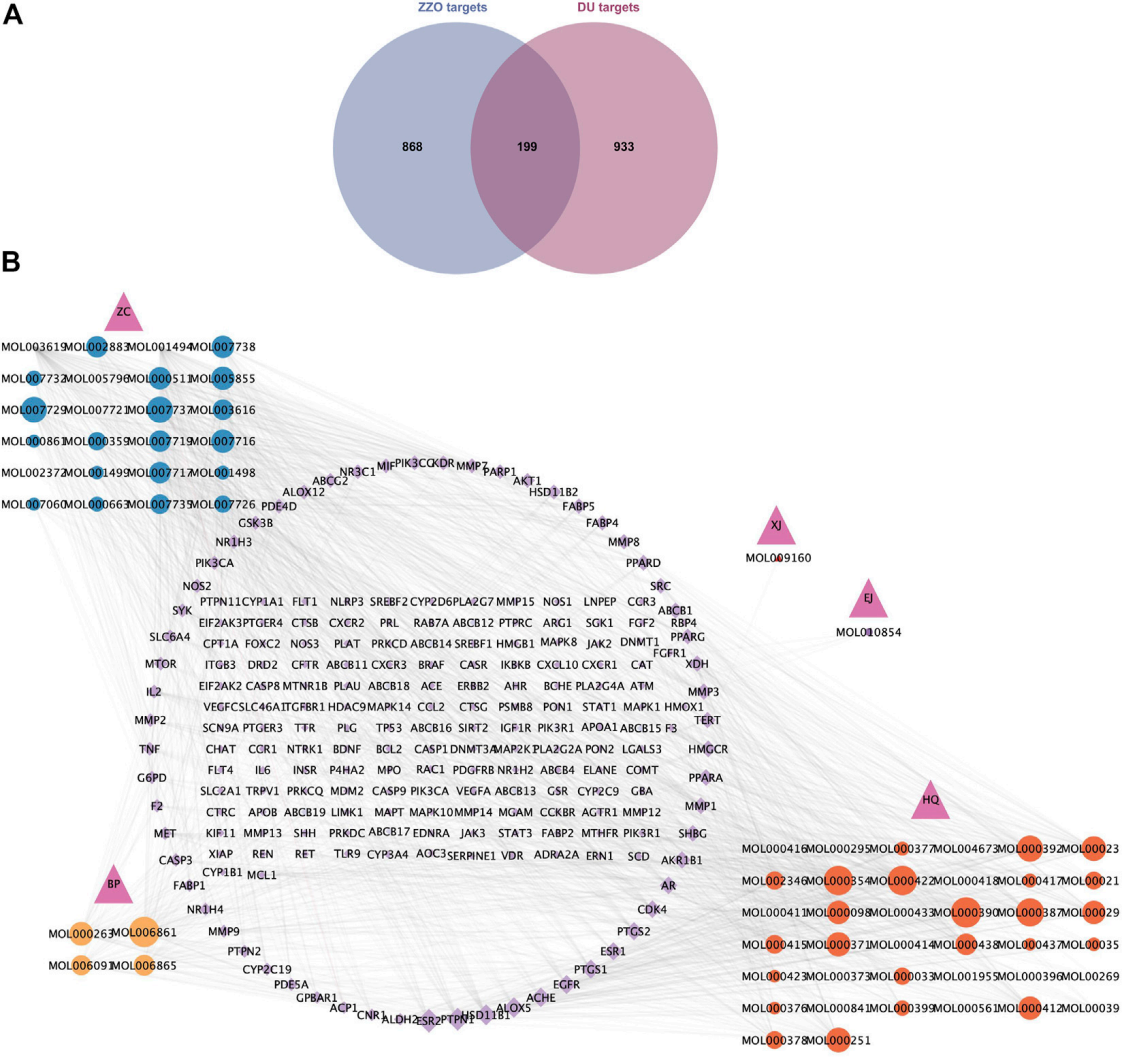
Common targets and C-T network of anti-DU ZZO. **(A)** Targets common to ZZO and DU. There are 199 common targets between ZZO and DU. **(B)** C-T network of ZZO anti-DU. Pink triangles represent herbal medicines, circles represent compounds, and diamonds represent targets. Node size reflects node degree: bigger size means a larger degree value.

Among the six active herb medicines, 1067 targets were retrieved from target prediction databases. A total of 1132 candidate targets for DU were obtained from the Genecard, Drugbank, and OMIM databases. Eventually, 199 common targets (Figure 3A) were considered potential targets of ZZO against DU. Then, the compound–target (C-T) network was constructed (Figure 3B). Among these bioactive components, isovalerylshikonin (ZC, degree = 97) exhibited the highest correlation with DU targets, and the other top four based on degree value were mandenol (ZC, degree = 65), daidzein (HQ, degree = 48), Asiatic acid (BP, degree = 48), and isorhamnetin (HQ, degree = 46). The details of the top ten compounds are summarized in Table 3.

**TABLE 3:** Information on top 10-degree compounds of ZZO.

No.	Compound	MOL ID	Molecular formula	Herb medicine	DL
1	Isovalerylshikonin	MOL003619	C_12_H_24_O_6_	Zicao	0.35
2	Mandenol	MOL001494	C_20_H_36_O_2_	Zicao	0.19
3	Daidzein	MOL000390	C_15_H_10_O_4_	Huangqi	0.19
4	Asiatic acid	MOL006861	C_30_H_48_O_5_	Bingpian	0.71
5	Isorhamnetin	MOL000354	C_12_H_12_O_7_	Huangqi	0.31
6	Kaempferol	MOL000422	C_15_H_10_O_6_	Huangqi	0.24
7	Bifendate	MOL000387	C_20_H_18_O_10_	Huangqi	0.67
8	α-Methyl-n-butylshikonin	MOL007737	C_21_H_28_O_5_	Zicao	0.35
9	Formononetin	MOL000392	C_16_H_12_O_4_	Huangqi	0.21
10	Shikonofuran C	MOL007729	C_21_H_26_O_5_	Zicao	0.3

### PPI network analysis

**FIGURE 4:**
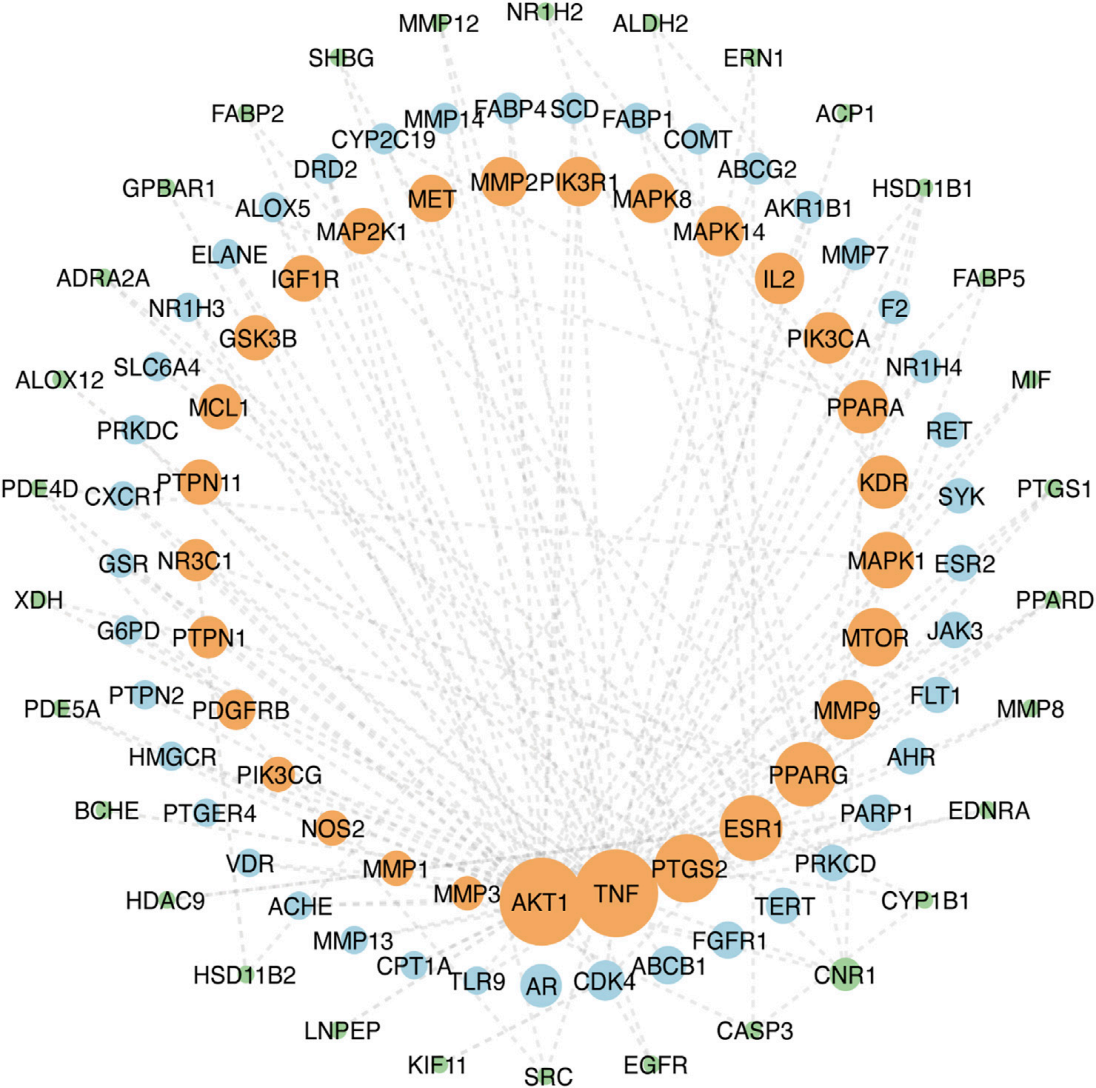
PPI network of ZZO anti-DU. Node size reflects node degree: bigger size means the larger degree value.

The top 100 common target genes were imported into the STRING database, and medium confidence of PPI was input to Cytoscape 3.9.1 for analyzing and constructing the PPI network (Figure 4). In the PPI network, targets with higher degrees played central roles in multi-protein interactions. The top 15 genes, ranked by degree value, were identified as the hub targets: *AKT1*(degree = 72), tumor necrosis factor (*TNF*; degree = 72), SRC proto-oncogene (*SRC*; degree = 57), epidermal growth factor receptor (EGFR; degree = 55), caspase 3 (*CASP3*; degree = 51), prostaglandin-endoperoxide synthase 2 (*PTGS2*; degree = 50), estrogen receptor (*ESR1*; degree = 47), peroxisome proliferator-activated receptor gamma (*PPARG*; degree = 46), matrix metallopeptidase 9 (*MMP9*; degree = 41), mechanistic target of rapamycin kinase (*MTOR*; degree = 40), mitogen-activated protein kinase 1 (*MAPK1*; degree = 38), kinase insert domain receptor (*KDR*; degree = 35), peroxisome proliferator-activated receptor alpha (*PPARA*; degree = 35), interleukin-2 (*IL-2*; degree = 33), and phosphatidylinositol-4,5-bisphosphate 3-kinase catalytic subunit alpha (*PIK3CA*; degree = 33).

### Pathway enrichment analysis of the candidate ZZO targets

**FIGURE 5:**
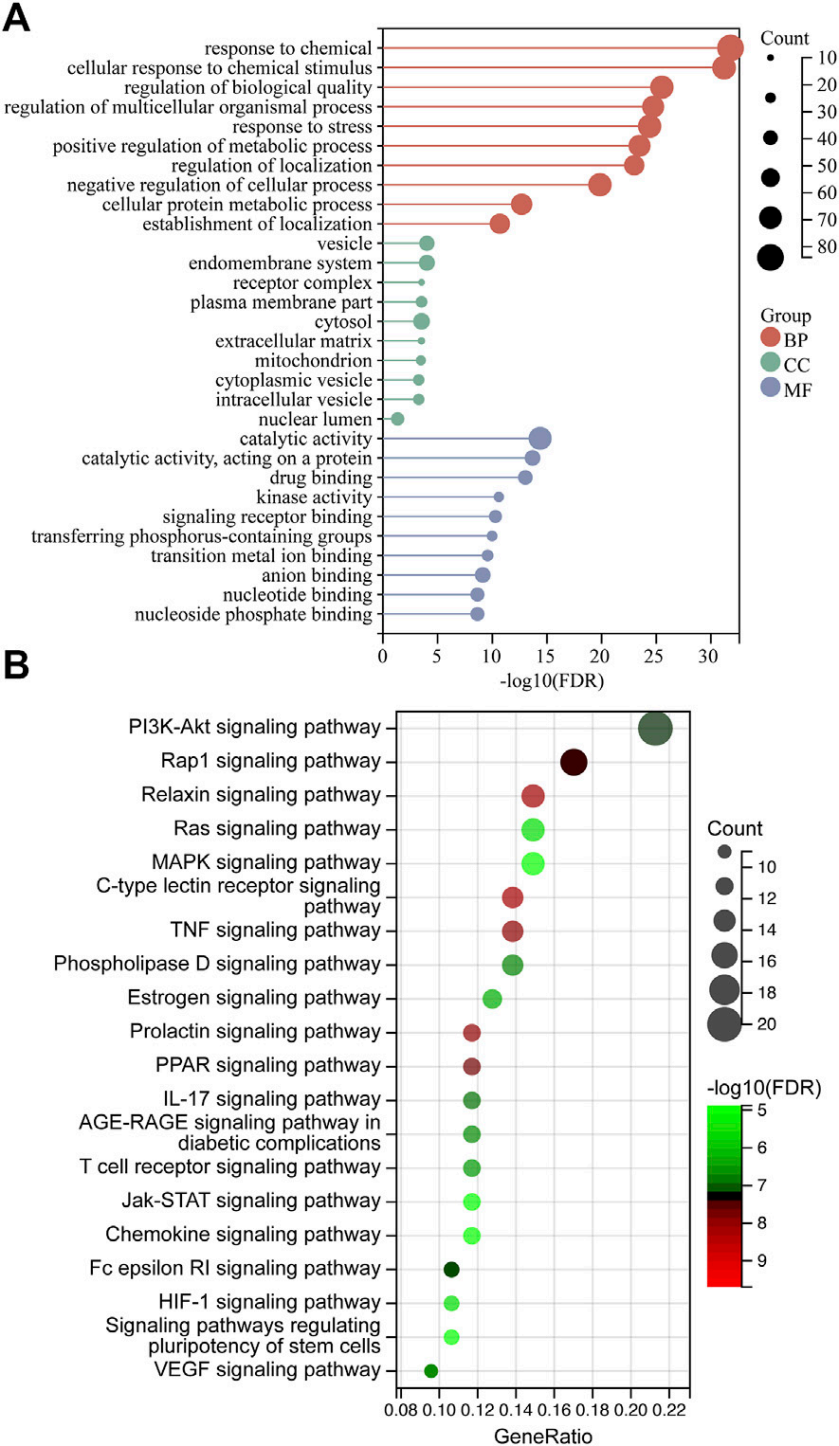
GO and KEGG analysis. **(A)** GO analysis of ZZO anti-DU. **(B)** KEGG analysis.

The 100-core putative ZZO targets were subjected to GO and KEGG analysis. The results revealed that 3380 biological process (BP), 251 cellular component (CC), and 3288 molecular function (MF) terms, and 136 pathways, were enriched among the targets (*p* < 0.05). The overall results of the GO analysis are summarized in Supplementary Table S3. The 10 significantly enriched BP, CC, and MF terms are visualized in Figure 5A. The GO analysis revealed that these targets are involved in response to chemicals, drugs, and cellular metabolic processes. After excluding human disease pathways, pathways with gene ratios >10 were visualized (Figure 5B); KEGG pathways are summarized in Supplementary Table S4. Various signaling pathways were linked to DU, especially the PI3K/AKT signaling pathway. This finding, in combination with a central protein analysis of the 100 core targets, indicates that AKT1 is the most important protein but that other proteins associated with the PI3K/AKT pathway are important as well, including EGFR, PIK3CG, KDR, PIK3CA, GSK3B, MTOR, IL-2, MET, MCL1, MAPK1, IGF1R, PDGFRB, MAPK8, MAPK1, PTGS2, and MMP9. Other important pathways are the TNF, MAPK, IL-17, chemokine, HIF, and VEGF signaling pathways, suggesting that ZZO anti-DU is highly correlated with anti-inflammation and pro-angiogenesis.

### Molecular docking of targets and compounds

**FIGURE 6:**
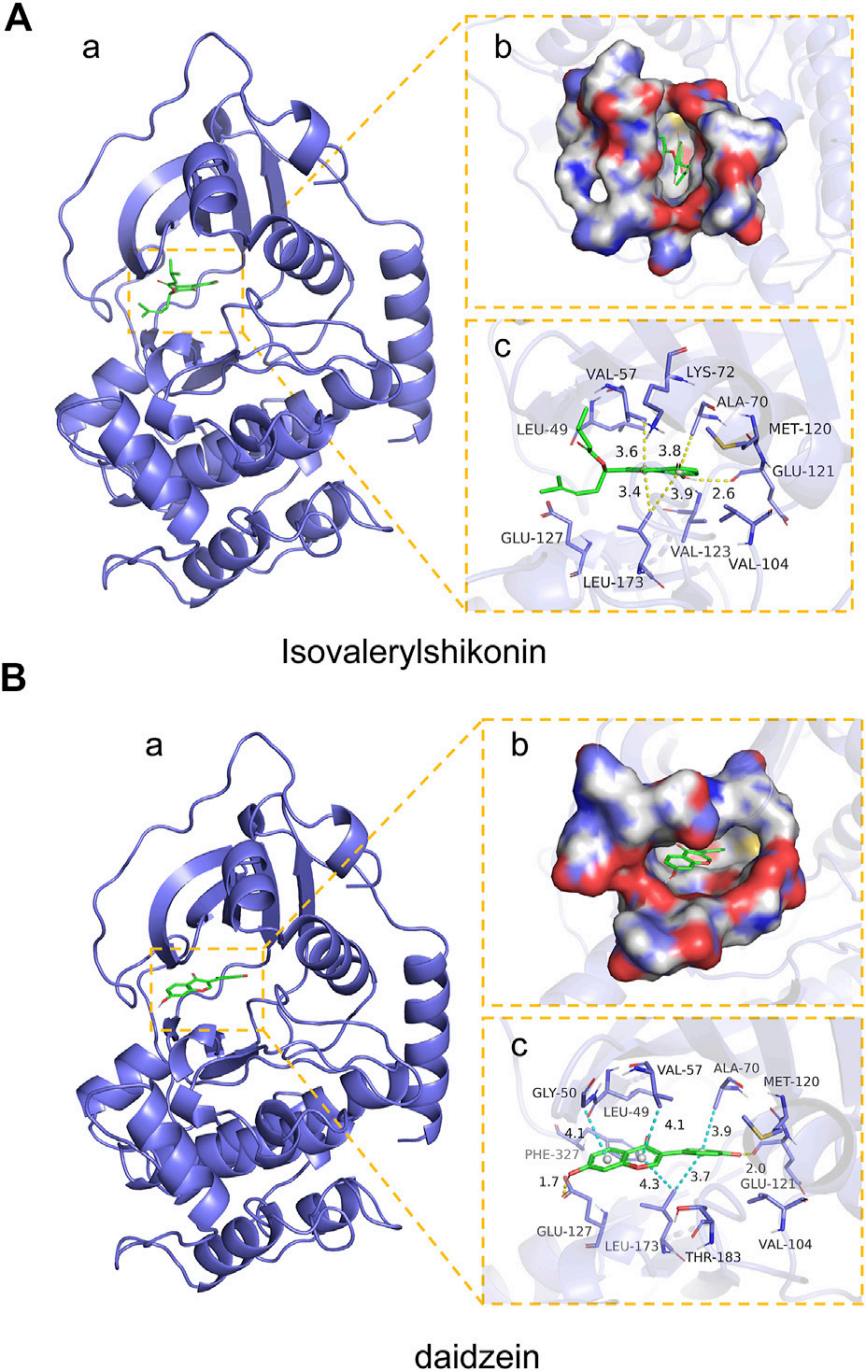
Mock docking of target and compounds. **(A)** Binding mode of AKT protein with ISO. (a) 3D structure of the complex. (b) Electrostatic surface of AKT protein. (c) Detailed binding mode of ISO with AKT protein. Yellow dashed lines represent hydrogen bonding interactions and blue dashed lines represent hydrophobic interactions. **(B)** Binding mode of AKT protein with Daidzein. (a) 3D structure of the complex. (b) Electrostatic surface of AKT protein. (c) Detailed binding mode of Daidzein with AKT protein. Yellow dashed lines represent hydrogen bonding interactions and blue dashed lines represent hydrophobic interactions.

We next analyzed the docking level of the top three-degree compounds (ISO, mandenol, and daidzein) with the AKT target. The results from the molecular docking software are shown in Table 4. Molecular docking results show that the conformation of ISO and daidzein with AKT showed good binding interactions, with binding energy of −7.09 kcal/mol and −7.81 kcal/mol, respectively. Then, using the Pymol2.1 software to visualize the binding process (Figure 6), ISO was shown to form a hydrogen bond with the hydroxyl group of (GLU-121) at the base of the AKT pocket and hydrophobic interaction with ALA-70, LEU-173, VAL-5 (Figure 6A). ISO could form stable complexes with AKT by these bindings. The hydrophobic groups of daidzein interact with the hydrophobic amino acids (ALA-70, LEU-173, VAL-57, GLY-50) at the base of the AKT pocket and play a role in stabilizing the compound–target network. Daidzein also combined with GLU-121 and GLU-127 by forming hydrogen bonding interactions with them (Figure 6B).

**TABLE 4:** Docking results for AKT with compounds.

Target ID	Compounds	Structure	Docking score (kcal/mol)	Combination type
AKT	Isovalerylshikonin	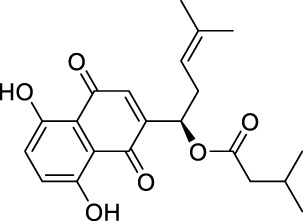	−7.09	Hydrogen bond Hydrophobic interactive
	Mandenol	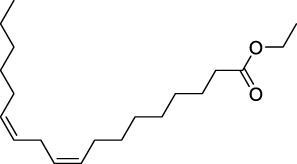	−3.26	Hydrophobic interactive
	Daidzein	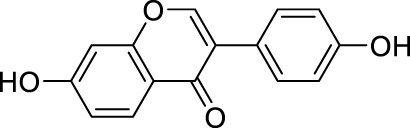	−7.81	Hydrogen bond Hydrophobic interactive

### ZZO promotes wound healing of DU

**FIGURE 7:**
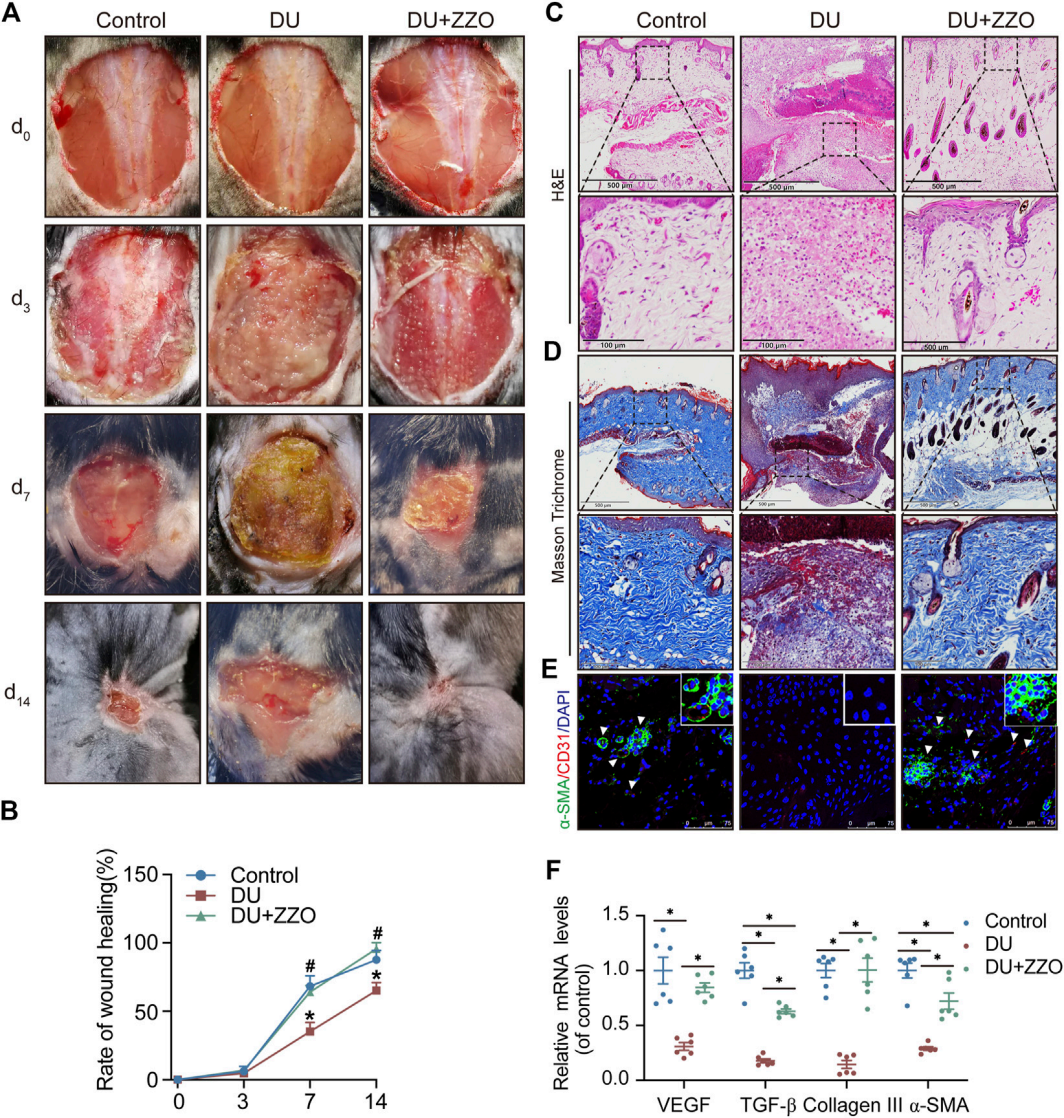
Efficacy of ZZO anti-DU *in vivo*. **(A,B)** Optical pictures and related quantification of the wound closure rate in the Control group, DU group, and DU + ZZO group at days 0, 3, 7, and 14 after the skin operation (*n* = 3, ^*^ *p* < 0.05, compared with the control group at the same time point, ^#^ *p* < 0.05, compared with the DU group at the same time point). **(C)** H&E staining images of wound tissues in the Control group, DU group, and DU + ZZO group at day 14 (*n* = 3 scale bar = 500 μm for 10× and 100 μm for 40×). **(D)** Masson’s trichrome staining at day 14 post-operation (*n* = 3, scale bar = 500 μm for 10× and 100 μm for 40×). **(E)** IF expression of *α*-SMA and CD31 in wound healing areas (*n* = 3, Scale bar = 75 μm). **(F)** Relative gene expression of the collagen synthesis-related genes *α-SMA*, *Collagen I*, and *Collagen III* in the Control group, DU group, and DU + ZZO group; relative gene expression of angiogenesis-related gene *VEGF* in the Control group, DU group, and DU + ZZO group (*n* = 6, *p < 0.05).

According to our results, ZZO significantly promoted wound healing of DU on days 7 and 14 (Figure 7A). The rate of wound healing was faster in the ZZO treatment group than in the DU group (Figure 7B). Additionally, H&E staining of the wound tissue revealed decreased inflammatory cells (i.e., polymorphonuclear leukocytes and plasma cells) and more integral epithelium in the ZZO group (Figure 7C). We also evaluated collagen deposition and angiogenesis in different groups. More collagen was formed, and the newly formed collagen fibers were remarkably thicker in the ZZO-treated group (Figure 7D). Colocation of *α*-SMA and CD31 revealed that the amount of newly formed blood vessels and collagen fibers were remarkably higher in the ZZO group compared with the DU group (Figure 7E). Meanwhile, the mRNA expression of *α-SMA*, *Collagen I*, *Collagen III*, and *VEGF* was higher in ZZO treatment group as well (Figure 7F). These results indicate that the treatment of ZZO promotes wound healing by anti-inflammation, pro-angiogenesis, and pro-fibrosis of the wound area in DU mice.

### ZZO promotes macrophage M2 polarization by activating PI3K/AKT signaling pathway in wound tissue of DU mice

**FIGURE 8:**
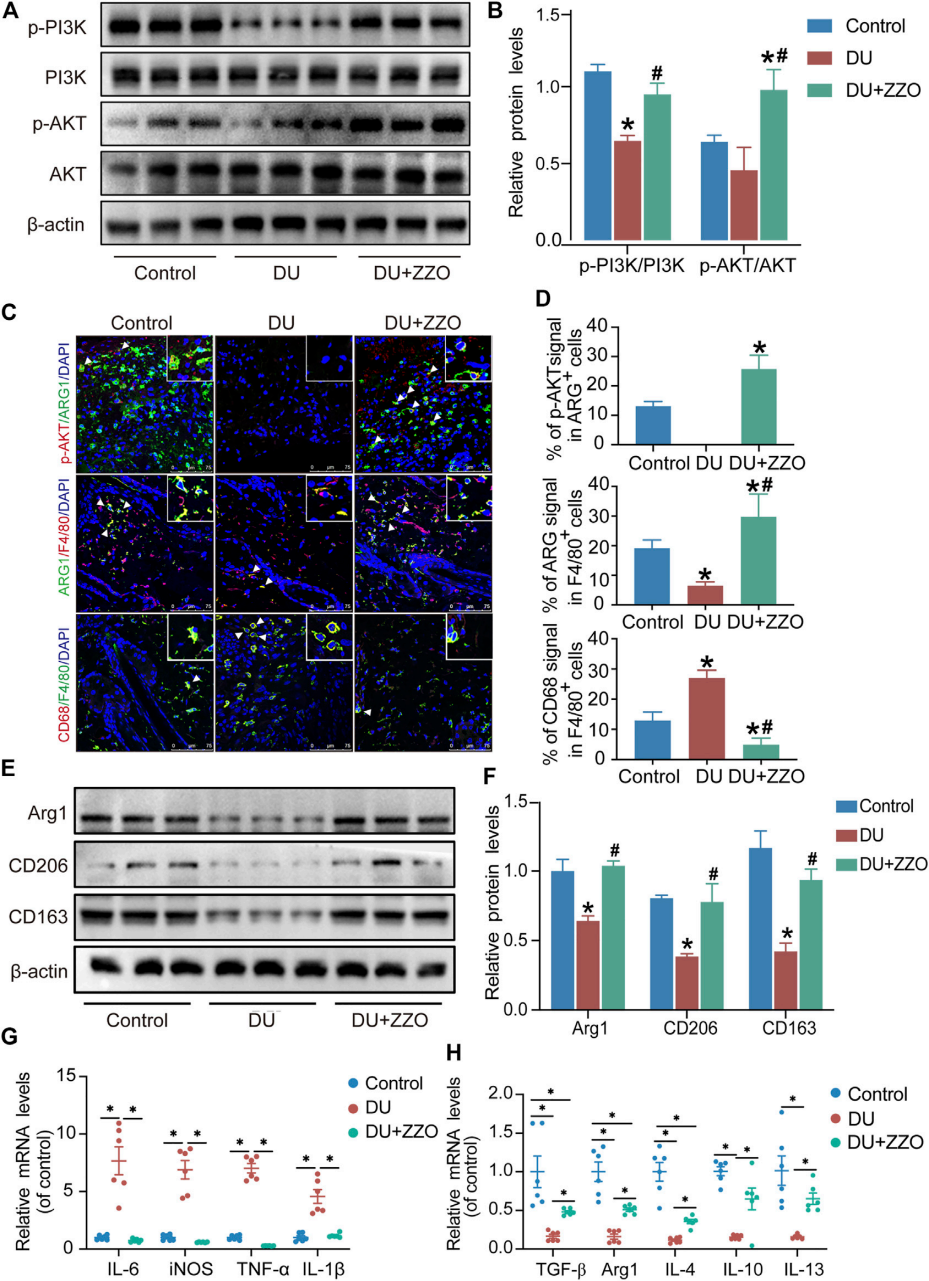
ZZO suppressed M1 macrophage-induced inflammation by activating the PI3K-AKT signaling pathway. **(A,B)** Expressions of PI3K, *p*-PI3K, AKT, and *p*-AKT proteins were tested in wound tissues (*n* = 3, ^*^ *p* < 0.05, compared with the control group, ^#^ *p* < 0.05, compared with the DU group). **(C,D)** Co-localization of *p*-AKT/ARG1, ARG1/F480, and CD68/F480 (*n* = 3, ^*^ *p* < 0.05, compared with the control group, ^#^ *p* < 0.05, compared with the DU group, Scale bar = 75 μm). **(E,F)** Expressions of ARG1, CD206, and CD163 proteins were tested in wound tissues (*n* = 3, ^*^ *p* < 0.05, compared with the control group, ^#^ *p* < 0.05, compared with the DU group). **(G)** mRNA expression of M1 macrophage markers (*n* = 6, *p < 0.05). **(H)** mRNA expression of M2 macrophage markers (*n* = 6, *p < 0.05).

**FIGURE 9:**
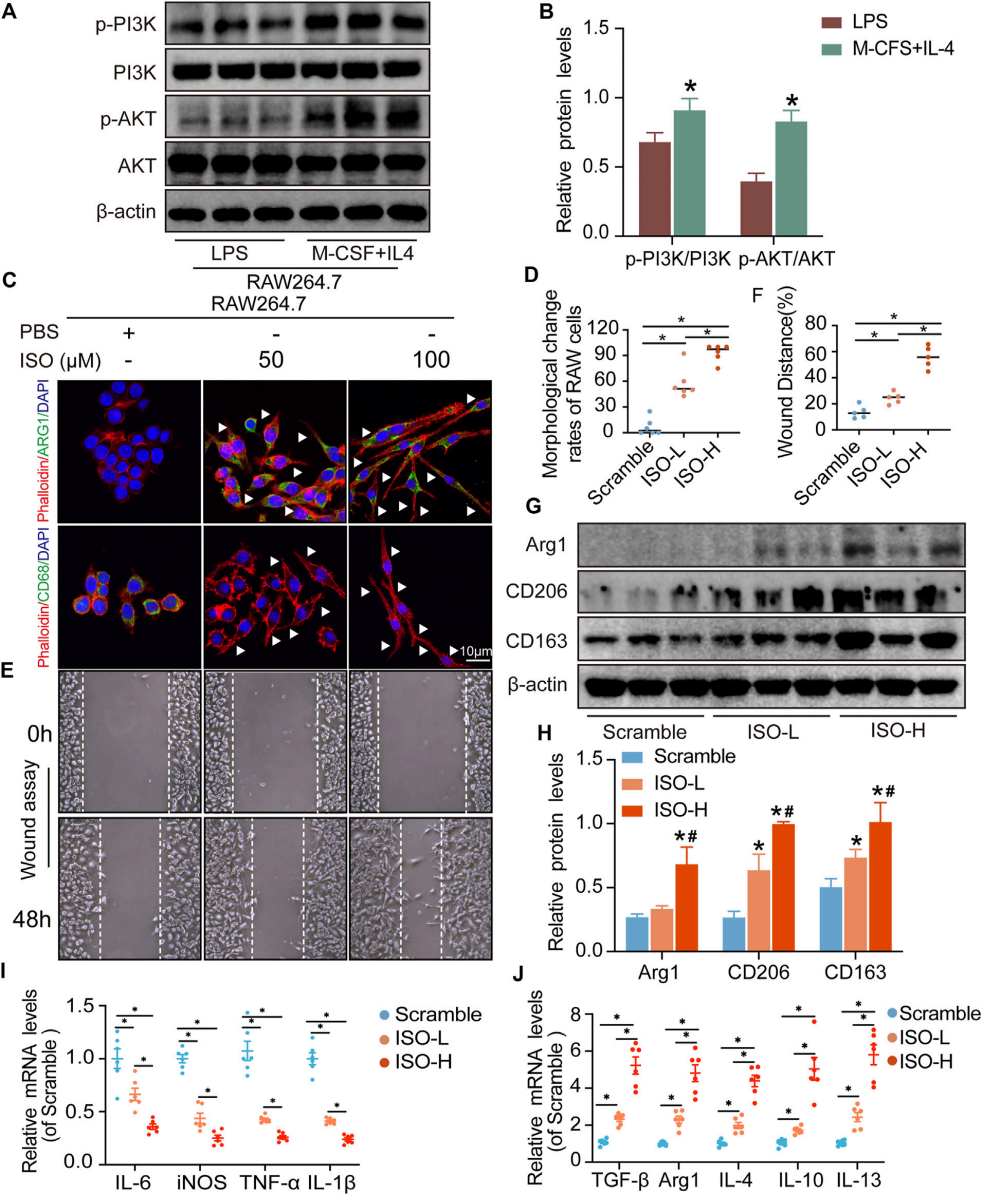
ISO promoted M2 polarization of macrophages *in vitro*. **(A,B)** Expressions of PI3K, *p*-PI3K, AKT, and *p*-AKT proteins were tested in RAW264.7 cells (*n* = 3, ^*^ *p* < 0.05, compared with the LPS group). **(C,D)** Visualization and quantitative analysis of co-location of phalloidin and CD68 or Arg1 of RAW264.7 cells in different groups (*n* = 6, ^*^ *p* < 0.05). **(E,F)** Visualization and quantitative analysis of wound assay of RAW264.7 cells in different groups (*n* = 6, ^*^ *p* < 0.05). **(G,H)** Expressions of ARG1, CD206, and CD163 proteins were tested on RAW264.7 cells in different groups. **(I)** mRNA expression of M1 macrophage markers (*n* = 3, *p < 0.05). **(J)** mRNA expression of M2 macrophage markers (*n* = 6, *p < 0.05).

The results of network analysis reveal that the PI3K/AKT signaling pathway is the most important pathway in ZZO anti-DU. Meanwhile, anti-inflammation pathways such as the TNF, IL-17, and chemokine signaling pathways play central roles as well. Therefore, we investigated the PI3K/AKT pathway and phenotypes of macrophages in wound tissues. The WB results showed that p-PI3K and p-AKT expression significantly decreased in diabetic mice, whereas those proteins were activated through ZZO treatment (Figures 8A,B). Furthermore, ZZO reversed diabetic-induced M1 macrophage infiltration and promoted M2 polarization of macrophages (Figures 8C–F). We also explored the co-expression of *p*-AKT and Arg1 in wound tissues, finding that the expression levels of *p*-AKT and Arg1 were positively correlated, which demonstrates that the M2 macrophage polarization in the ZZO group may be related to activation of the PI3K/AKT pathway (Figure 8C). We further induced M1 polarization by LPS and M2 polarization by M-CSF and IL4 in RAW264.7 cells. Indeed, M2 macrophages showed strong activation of the PI3K/AKT pathway (Figure 9A). These results indicate that diabetic mice show an inflammatory microenvironment in wound tissues but that ZZO could alleviate this environment by recruiting M2 macrophages to activate the PI3K/AKT pathway.

### ISO treatment promotes M2 macrophage polarization *in vitro*

The results of network analysis show that ISO, mandenol, and daidzein are the most important active anti-DU compounds of ZZO. We further conducted molecular docking studies to find a suitable tight ligand binding compound. ISO forms a strong tight ligand with AKT (Figure 6A). Therefore, we constructed a series of ISO-stimulated *in vitro* experiments in RAW264.7 cells. ISO changed the morphology of RAW264.7 cells, which transitioned from the M1 to the M2 phenotype (Figures 9C,D). Moreover, ISO prompted the invasion of RAW264.7, indicating an M2 phenotype (Figures 9E,F). The protein expression of ARG1, CD206, and CD163 all increased after ZZO treatment (Figures 9G,H). Furthermore, we detected the levels of proinflammatory (*iNOS*, *TNF-*α, *IL-6*, and *IL-1*β), as well as anti-inflammatory factors (*IL-4*, *IL-13*, *IL-10*, *TGF-β*, and *ARG1*) by RT-PCR, and the results fully correspond with the *in vivo* findings (Figures 9I,J).

## Discussion

DU is a primary complication of diabetes and has always been a health issue in long-term clinical practice. Wound healing in DU is a prolonged process with various stages, including hemostasis, inflammation, proliferation, and remodeling (Zarei et al., 2018). Diverse cells, with different chemokine activities, take part in the process of wound healing at different stages of DU (Zhao et al., 2016). During the inflammation stage, wound tissue harbors predominantly macrophages, which both promote and inhibit inflammation (Louiselle et al., 2021). In routine wound healing, M2 macrophages occupy the wound healing area with fewer M1 macrophages existing after three days, whereas M1 macrophages continue to be present in the wound area, with little or even no M2 macrophages present in the DU site (Louiselle et al., 2021). During the proliferative stage, wound healing features granulation tissue formation, collagen synthesis, and angiogenesis, while diabetes promotes the production of advanced glycation end products, which inhibit the proliferation and migration of fibroblasts (Li et al., 2017). Due to these various cell types, cell–cell interactions, chemokines, and complex mechanisms, it is difficult to achieve the desired therapeutic effect of DU.

The multicomponent–multitarget methods of TCM offer various therapeutic activities for DU. As discussed in the introduction, our previous studies have verified that ZZO can promote wound healing in DU though the specific compounds and potential mechanisms have remained unclear. Thus, we adopted network analysis to determine the active compounds and anti-DU mechanisms of ZZO. The results show that ZC and HQ play key roles in anti-DU ZZO. After excavating related targets, the PI3K/AKT pathway was identified for further study. PI3K is activated under physiological stimuli and binds to receptor tyrosine kinase provided with a ligand binding domain. A central component in the PI3K pathway is the serine/threonine-specific protein kinase AKT, with multiple downstream target proteins (Madhunapantula et al., 2011). The PI3K/AKT cascade especially contributes to macrophage polarization (Tan et al., 2018), and *PI3K* knockout (KO) mice induce NF-κB activation and IL-1β release, which is characteristic of the M1 phenotype (Solinas and Becattini, 2017). Our results also show activation of PI3K/AKT in M2 macrophages. Thus, we constructed a DU mouse model and evaluated the condition of the PI3K/AKT signaling pathway. In DU mice, the PI3K/AKT pathway was inhibited and prone to greater M1 macrophage infiltration in wound tissues. Surprisingly, the PI3K/AKT pathway was activated with M2 macrophage infiltration after ZZO treatment.

Moreover, M2 macrophages can recruit fibroblasts directly or by the secretion of chemokines, such as TGF-β, which promote collagen deposition and damage repair (Jie et al., 2018). Meanwhile, during the angiogenesis stage, macrophages induce blood vessel maturation through the secretion of VEGF and IGF-1 (Gurevich et al., 2018). Altogether, M2 macrophages promote fibroblast cell proliferation, ECM reconstruction, and angiogenesis. Thus, we found that treatment of ZZO facilitates the secretion of IL-4, IL-10, and VEGF in macrophages, thereby inducing fibrosis and angiogenesis during wound healing. Furthermore, the autodocking results show that ISO is the highest-binding compound for AKT. Thus, we treated RAW264.7 cells with ISO to observe their phenotype *in vitro*. Similarly, ISO promotes the transition of RAW264.7 cells into the M2 phenotype in a dose-dependent manner.

**FIGURE 10:**
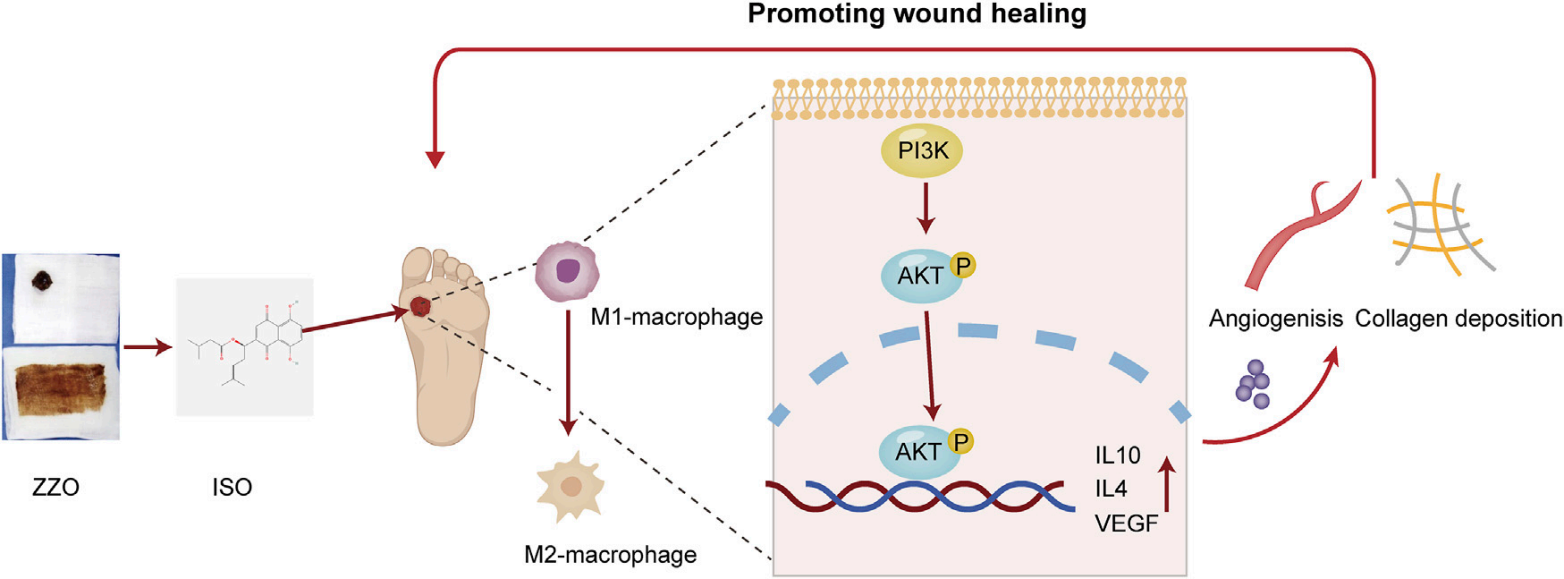
Mechanisms of ZZO anti-DU. ZZO could promote M2 polarization by activating the PI3K/AKT pathway and then secreting IL-4, IL10, and VEGF to promote collagen deposition and angiogenesis. These processes help wound healing faster in DU mice.

In conclusion, we conducted an integrative network analysis and experimental evaluation of ZZO against DU. We evaluated the activation of the PI3K/AKT pathway under ZZO treatment. We found that ZZO can promote M2 polarization by activating the PI3K/AKT pathway and then secreting IL-4, IL10, and VEGF to promote collagen deposition and angiogenesis (Figure 10). However, the network analysis has its own limitations. The data is derived from public databases, which are constantly updated; therefore, some other bioactive compounds may have been excluded. Moreover, targets and pathways that come from network analysis may lead to bias in the study. Considering this, PI3K/AKT inhibitors and activators may be required to further verify this mechanism. Moreover, the complex components and mechanisms of ZZO may affect the promotion of its clinical application in the future. In the future, we will focus more on the nano-gel formulation of single compounds, such as ISO, and anticipate promoting their clinical efficacy in DU.

## Data Availability

The datasets presented in this study can be found in online repositories. The names of the repository/repositories and accession number(s) can be found in the article/Supplementary Material.
